# Multi-Target Detection Method Based on Variable Carrier Frequency Chirp Sequence

**DOI:** 10.3390/s18103386

**Published:** 2018-10-10

**Authors:** Wei Wang, Jinsong Du, Jie Gao

**Affiliations:** 1 Shenyang Institute of Automation, Chinese Academy of Sciences, Shenyang 110016, China; wangwei2@sia.cn (W.W.); gaojie@sia.cn (J.G.); 2 Institutes for Robotics and Intelligent Manufacturing, Chinese Academy of Sciences, Shenyang 110016, China; 3 University of Chinese Academy of Sciences, Beijing 100049, China

**Keywords:** multi-target detection, continuous wave radar systems, variable carrier frequency chirp sequence, Doppler ambiguity

## Abstract

Continuous waveform (CW) radar is widely used in intelligent transportation systems, vehicle assisted driving, and other fields because of its simple structure, low cost and high integration. There are several waveforms which have been developed in the last years. The chirp sequence waveform has the ability to extract the range and velocity parameters of multiple targets. However, conventional chirp sequence waveforms suffer from the Doppler ambiguity problem. This paper proposes a new waveform that follows the practical application requirements, high precision requirements, and low system complexity requirements. The new waveform consists of two chirp sequences, which are intertwined to each other. Each chirp signal has the same frequency modulation, the same bandwidth and the same chirp duration. The carrier frequencies are different and there is a frequency shift which is large enough to ensure that the Doppler frequencies for the same moving target are different. According to the sign and numerical relationship of the Doppler frequencies (possibly frequency aliasing), the Doppler frequency ambiguity problem is solved in eight cases. Theoretical analysis and simulation results verify that the new radar waveform is capable of measuring range and radial velocity simultaneously and unambiguously, with high accuracy and resolution even in multi-target situations.

## 1. Introduction

**Figure 1:**
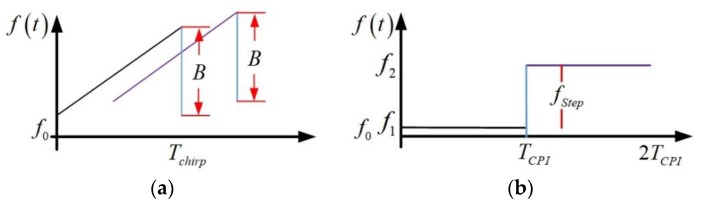
Two typical waveforms (**a**) Linear Frequency Modulation (LFM) signal and (**b**) Frequency Shift Keying (FSK) signal.

With the development of electronic technology, the application field of radar has expanded to the fields of intelligent transportation systems [1,2], vehicle assisted driving [3,4], positioning and navigation [5], health monitoring [6], pedestrian and obstacle detection [7,8], etc. The selection of waveform type and signal processing technology depends to a great extent on the specific tasks and functions of radar. Civilian fields, such as intelligent transportation and automotive collision detection, require radar to have the characteristics of simple structures, low cost and high integration [9,10], thus the continuous wave system is the preferred. There are several continuous waveform (CW) proposals which have been developed in the last years for new radar systems to measure range and radial velocity simultaneously. Linear Frequency Modulation (LFM) [11,12] and Frequency Shift Keying (FSK) [13] which are shown in Figure 1 are two typical waveforms.

The received signal of LFM is directly mixed to baseband by the transmitted signal. Therefore, the resulting baseband beat frequency $f_{B}$ describes the difference between the transmitted frequency and the received frequency. In the situation of stationary target, the propagation delay leads to frequency shift $f_{R}$, and the beat signal frequency is influenced by the target distance R only. However, when the target is moving, the beat signal frequency depends on the target range $f_{R}$ and the Doppler frequency $f_{D}$, as shown in Equation (1).

(1) $$f_{B}=f_{R}-f_{D}=-\frac{B}{T_{chirp}}\frac{2}{c}R+\frac{2}{\lambda }v$$

where *B* is the frequency modulation bandwidth, *T_chirp_* is the frequency modulation period and the distance and speed of the target can be solved by the up beat frequency and the down beat frequency. However, in the case of multiple targets, there are many errors in combinations of up beat frequencies and down beat frequencies. In order to solve the distance-velocity coupling problem, multiple sets of chirped continuous waves with different frequency modulations are needed. The extended measurement time is an important drawback of this LFM technique.

FSK system transmits two discrete frequencies $f_{1}$ and $f_{2}$ sequentially with a duration of $T_{CPI}$. The frequency step between the two carriers is represented as $f_{Step}=f_{2}-f_{1}$. The received signal is converted to baseband signal by a homodyne receiver. The baseband outputs carry the Doppler frequencies generated by the moving target. By maintaining $f_{Step}$ very small in comparison to the transmitted signals, the Doppler frequencies extracted from the baseband outputs will be approximately the same. The peak phase difference is used to estimate the distance, as shown in Equation (2).

(2) $$R=-\frac{c\cdot \Delta \varphi }{4\pi \cdot f_{Step}}$$

It can be realized easily, which is the advantage of FSK waveform. However, when multiple targets are moving at the same speed or when multiple targets are stationary, multiple targets cannot be distinguished by phase information, which is the inherent defect of FSK waveform. The combination of FSK and LFM waveform design principle offers the possibility of an unambiguous target range and velocity measurement simultaneously [14,15,16,17]. Multi Frequency Shift Keying (MFSK) consist in this case of two linear frequency modulated up-chirp signals. However, this technique suffers from low estimation accuracy due to the phase measurement.

Chirp Sequence (CS) radars are gaining popularity because of their inherent ability to extract the range and velocity parameters of multiple targets from the beat signals based on two-dimensional fast Fourier transform [18,19,20,21]. However, the chirp sequence waveform also has a drawback; that is, the maximum unambiguously measurable velocity is limited by the repetition interval of the chirps. The Doppler ambiguity problem occurs when the velocity of a target exceeds the maximum measurable velocity [22]. The maximum unambiguous frequency can be increased by increasing the chirp repetition rate of the chirp sequence (reducing the duration of the chirp sequence). The signal needs to meet a certain number of sampling points, so the sampling frequency should be increased, and the hardware should meet the data processing rate. Therefore, the above methods still cannot effectively solve the current problem. Multiple approaches have been developed to resolve Doppler ambiguity [23,24,25,26]. A modified chirp sequence waveform was developed by adding an additional frequency shift between every two adjacent chirps [23,24]. Doppler ambiguities can be resolved using the additional phase information introduced by the frequency shift. Frequency shift is a key factor in determining the phase difference. It is difficult to maintain high-precision frequency shift. The frequency shift between every two adjacent chirps is changed to delay. The delay of the new improved waveform can be precisely controlled by the system clock. The waveform implementation is simpler. Moreover, the accuracy of the resolved unambiguous Doppler frequencies is not well guaranteed since phase information is susceptible to noise interference.

Therefore, the principle of waveform design for continuous wave radar includes the following points:

(1) Waveforms must have probing capabilities for multi-target scenes to meet the application requirements of intelligent transportation, automotive collision detection, etc.
(2) Under current conditions, it is difficult to meet the accuracy requirements by extracting the phase information to solve the parameters such as velocity and range. Waveforms must be simple in form and easy to implement in hardware.
(3) It is not possible to increase the computational complexity and increase the cost of hardware because of the special signal form.

The main contribution of this paper is to design a waveform that satisfies the above principles. The new radar waveform, which is improved on the chirp sequence, is capable of measuring range and radial velocity simultaneously and unambiguously, with high accuracy and resolution even in multi-target situations. The remainder of this paper is organized as follows. Section 2 introduces the chirp sequence waveform and its corresponding signal model, and analyzes the Doppler ambiguity problem. In Section 3, the variable carrier frequency chirp sequence waveform and its corresponding signal processing method are proposed. Simulation results, which are shown in Section 4, verify that the new radar waveform has the adaptability for multi target situations. Section 5 provides some conclusions.

## 2. Chirp Sequence Waveform

**Figure 2:**
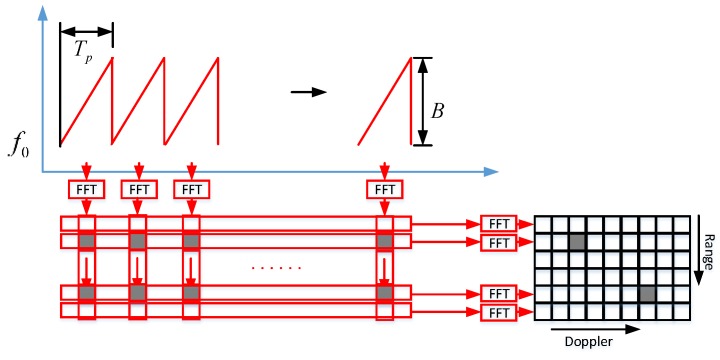
2D-FFT processing based on chirp sequence.

The classical chirp sequence waveform is shown in Figure 2. The waveform consists of L equispaced chirps. Each single chirp has a duration of $T_{p}$. This baseband signal is described by the following equation:

(3) $$s\left(t,l\right)=\exp\left(j2\pi \left(f_{B}\cdot t-f_{D}\cdot l\cdot T_{p}+\varphi \right)\right)$$

where parameter l describes the chirp number. The parameter φ describes the phase. The beat frequency $f_{B}$ contains both the target range $f_{R}$ and Doppler frequency $f_{D}$ (see Equation (1)).

This baseband signal s(t,l) is sampled and FFT processed for each individual chirp signal, which splits the echo signal into $N_{FFT}$ adjacent range gates. Then the Doppler FFT is performed in each range gate. The range–Doppler spectrum is stored as a matrix $S_{RD}$ of $N_{FFT}$ rows and $L_{FFT}$ columns. The parameter $N_{FFT}$ and the parameter $L_{FFT}$ represent the FFT length. The process is shown in Figure 2.

The target information can be extracted from the matrix $S_{RD}$. The beat frequency $f_{B}$ and the Doppler frequency $f_{D}$ are shown in the following equation:

(4) $$f_{B}=f_{R}-f_{D}=-\frac{B}{T_{p}}\frac{2}{c}\cdot R+2\frac{v}{\lambda }$$

(5) $$f_{D}=-2\frac{v}{\lambda }$$

Based on this chirp sequence waveform, radar targets are resolved in range and in radial velocity separately.

(6) $$R=-\left(f_{B}+f_{D}\right)\cdot \frac{T_{p}}{B}\frac{c}{2}$$

(7) $$v=-f_{D}\frac{\lambda }{2}$$

According to the Shannon/Nyquist sampling theorem, aliasing occurs when the sampling rate is less than two times the maximum frequency. Therefore, the Doppler ambiguity problem will occur when the chirp repetition rate of the chirp sequence waveform is lower than two times that of the maximum Doppler frequency. The maximum unambiguous Doppler frequency is denoted by the following equation:

(8) $$f_{D,\max}=f_{r}/2=1/\left(2\cdot T_{p}\right)$$

The Doppler frequency detected by the range–Doppler spectrum is $f_{D,amb}$, and the actual Doppler frequency $f_{D}$ can be obtained by adding a multiple q of the maximum unambiguous Doppler frequency $2f_{D,\max}$ to $f_{D,amb}$:

(9) $$f_{D}=f_{D,amb}+q\cdot \left(2f_{D,\max}\right)$$

where q represents the number of Doppler frequency aliasing.

**Figure 3:**
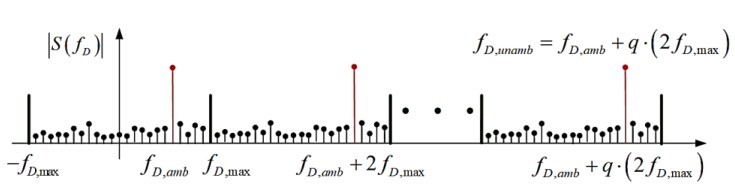
Doppler ambiguity schematic.

The unknown factor of q represents the ambiguity of the Doppler frequency measurement, as depicted in Figure 3. Assuming that the duration of the chirp sequence is 0.1 ms, the repetition frequency of the chirp signal $f_{r}$ = 1000 Hz. This means that for the slow time domain, the sampling frequency is 1000 Hz. The Doppler frequency range by the second FFT is from −500 Hz to 500 Hz. We assumed that the carrier frequency $f_{0}$ is 24 GHz. According to the relationship between velocity and Doppler frequency, the maximum unambiguous velocity can be calculated using the following equation:

(10) $$v_{unamb\max}=-\frac{f_{D,\max}c}{2f_{0}}$$

The unambiguous velocity is from −11.25 km/h to 11.25 km/h. Obviously, it does not meet the needs of intelligent transportation and automotive collision avoidance. The maximum unambiguous frequency can be increased by increasing the chirp repetition rate of the chirp sequence (reducing the duration of the chirp sequence). Assuming that the duration of the chirp sequence is 0.01 ms, the maximum unambiguous frequency is 5000 Hz. The maximum unambiguous velocity is from −112.5 km/h to 112.5 km/h. The signal needs to meet a certain number of sampling points, so the sampling frequency should be increased, and the hardware should meet the data processing rate. Therefore, the above methods still cannot effectively solve the current problem. Doppler ambiguity is a significant disadvantage of the chirp sequence.

## 3. Variable Carrier Frequency Chirp Sequence

The new waveform consists of two chirp sequences, which are intertwined to each other. The length of the two intertwined waveforms is $2LT_{p}$. Each chirp signal has the same frequency modulation, the same bandwidth B and the same chirp duration $T_{p}$. The carrier frequencies are $f_{01}$ and $f_{02}$ respectively. The frequency shift $f_{shift}$ is the difference between frequency $f_{01}$ and $f_{02}$, that is, $f_{shift}=f_{02}-f_{01}$ ($f_{02}>f_{01}$). In the paper [24], the frequency shift $f_{shift}$ is so small that the beat frequency measurement and ambiguous Doppler frequency measurement are not influenced by this frequency shift waveform parameter. The frequency shift in this paper is large enough to ensure that the Doppler frequencies in the two-chirp sequence for the same moving target are different.

The baseband signals are described by the following equations:

(11) $$s_{1}\left(t,l\right)=\exp\left(j2\pi \left(f_{B1}\cdot t-f_{D1}\cdot 2l\cdot T_{p}+\varphi_{1}\right)\right)$$

(12) $$s_{2}\left(t,l\right)=\exp\left(j2\pi \left(f_{B2}\cdot t-f_{D2}\cdot \left(2l+1\right)\cdot T_{p}+\varphi_{2}\right)\right)$$

**Figure 4:**
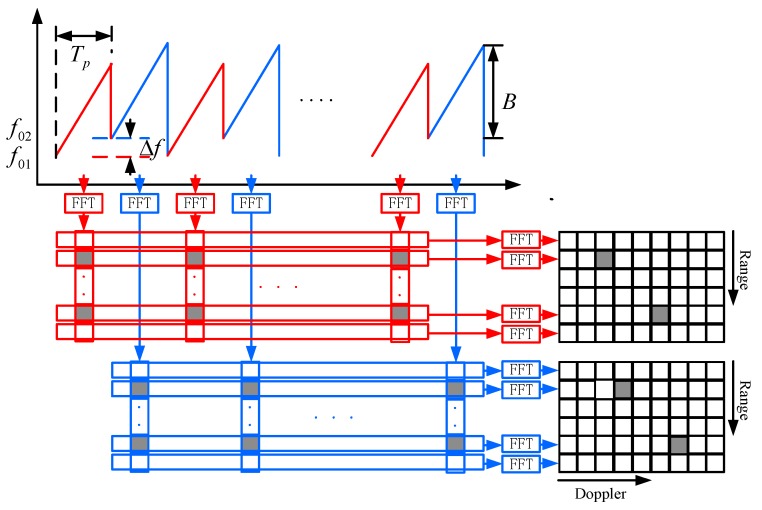
2D-FFT processing based on variable carrier frequency chirp sequence.

The two time-discrete signals are processed by a first FFT to calculate the beat frequency $f_{B}$ and by a second FFT to measure the Doppler frequency $f_{D,amb}$. The range–Doppler spectrums are stored as matrix $S_{RD1}$ and $S_{RD2}$. The process flow of the variable carrier frequency chirp sequence is similar to the chirp sequence, as shown in Figure 4.

For the range–Doppler spectrum $S_{RD1}$,

(13) $$f_{B1}=f_{R}-f_{D1}=-\frac{B}{T_{p}}\frac{2}{c}\cdot R+2\frac{v}{\lambda_{1}}$$

(14) $$f_{D1}=f_{D1,amb}+q_{1}\cdot \left(2f_{D,\max}\right)=-2\frac{v}{\lambda_{1}}=-2\frac{v}{c}f_{01}$$

For the range–Doppler spectrum $S_{RD2}$,

(15) $$f_{B2}=f_{R}-f_{D2}=-\frac{B}{T_{p}}\frac{2}{c}\cdot R+2\frac{v}{\lambda_{2}}$$

(16) $$f_{D2}=f_{D2,amb}+q_{2}\cdot \left(2f_{D,\max}\right)=-2\frac{v}{\lambda_{2}}=-2\frac{v}{c}f_{02}$$

For the same moving target, the Doppler frequencies in the two-chirp sequence are different due to the different carrier frequencies.

(17) $$\Delta f_{D}=f_{D2}-f_{D1}=-\left(2\frac{v}{c}\left(f_{02}-f_{01}\right)\right)=-2\frac{v}{c}f_{shift}$$

For the target P, the row and column indices of the matrix $S_{RD1}$ and the matrix $S_{RD2}$ are ($n_{1}^{P}$,$l_{1}^{P}$) and ($n_{2}^{P}$,$l_{2}^{P}$). Where $n_{1}^{P}$ and $n_{2}^{P}$ correspond to the beat frequencies $f_{B1}$ and $f_{B2}$, $l_{1}^{P}$ and $l_{2}^{P}$ correspond to the Doppler frequencies $f_{D1,amb}$ and $f_{D2,amb}$ (possibly frequency aliasing). The Doppler frequencies $f_{D1}$ and $f_{D2}$ are very close, so the influence on the beat frequencies $f_{B1}$ and $f_{B2}$ is small. The frequency resolution of the range domain is on the order of 10^2^ Hz. Therefore, $n_{2}^{P}$ is usually equal to $n_{1}^{P}$. The frequency resolution of the Doppler domain is on the order of 0.1 Hz. Then, $l_{1}^{P}$ and $l_{2}^{P}$ show differences.

The velocity of the target can be calculated through the following equation:

(18) $$v=-\left[f_{D2,amb}-f_{D1,amb}+\left(q_{2}-q_{1}\right)\left(\pm 2f_{D,\max}\right)\right]c/\left(2f_{shift}\right)$$

where $f_{D,\max}=1/\left(2\cdot 2T_{p}\right)$ represents the largest unambiguous frequency, because the repetition frequency of the new signal $f_{r}$ is $1/\left(2T_{p}\right)$. The symbol of $f_{D,\max}$ is determined according to the aliasing direction. $q_{2}$ and $q_{1}$ indicate the number of Doppler frequency aliasing. 

According to the sign and numerical relationship of the Doppler frequency $f_{D1,amb}$ and $f_{D2,amb}$, the calculation of the Doppler frequency difference is divided into eight cases, which are shown in Table 1.

Calculate the Doppler frequency difference $\Delta f_{D}$ according to Table 1. Then the radial velocity of the target can be calculated as follows:

(19) $$v=-\Delta f\cdot c/\left(2f_{shift}\right)$$

Although the frequency values of $f_{D1,amb}$ and $f_{D2,amb}$ are very accurate, the error of the Doppler frequency difference $\Delta f_{D}$ is small. However, when the radial velocity is calculated according to Equation (19), the error of the radial velocity is amplified. Therefore, it is necessary to correct the velocity value.

The parameter q is calculated as follows:

(20) $$q_{1}=round\left\{\frac{f_{D1,u}-f_{D1,amb}}{2f_{D,\max}}\right\}$$

where the Doppler frequency $f_{D1,u}$ can be calculated according to Equation (14). 

Recalculate the Doppler frequency $f_{D1}$ and the radial velocity according to the following equations:

(21) $$f_{D1}=f_{D1,amb}+q_{1}\cdot \left(2f_{D,\max}\right)$$

(22) $$v_{r}=-\frac{c\cdot f_{D1}}{2f_{01}}$$

Then calculate the range of the target according to Equation (13):

(23) $$R=-\left(f_{B1}-2\frac{v}{\lambda_{1}}\right)\frac{T_{p}\cdot c}{2B}$$

**Figure 5:**
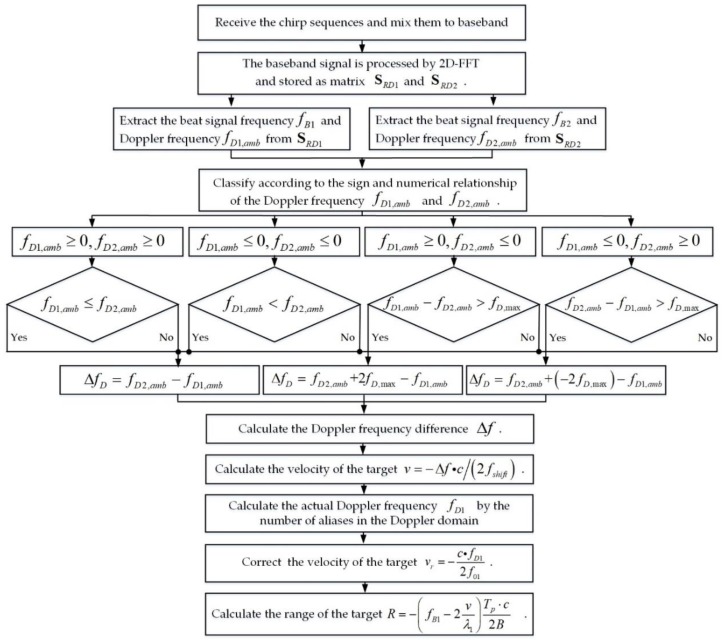
Multi-target detection method based on variable carrier frequency chirp sequence.

The overall flow of the multi-target detection method is shown in Figure 5.

## 4. Simulation

In this section, the simulation results verify the validity of waveform in its signal processing method. The basic parameters of the variable carrier frequency chirp sequence are shown in Table 2.

**Figure 6:**
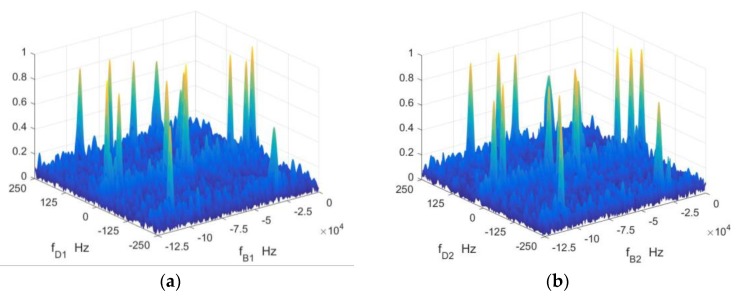
The range-Doppler spectrum (**a**) first set of chirp sequences and (**b**) second set of chirp sequences.

**Figure 7:**
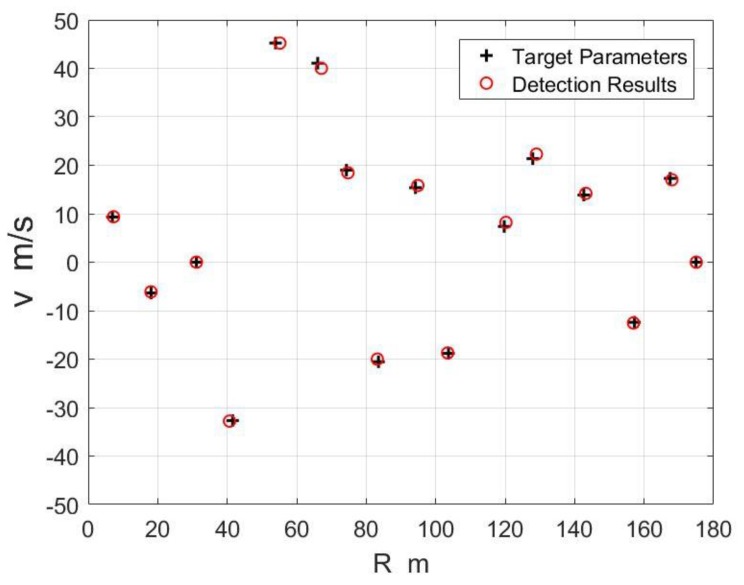
The detection results of the targets.

In order to verify the validity of the waveform in multi target situations, 16 point targets are simulated. The signal-to-noise ratio of the time domain signal is 0 dB. The amplitude spectrums of the baseband signals of two sets of chirp sequences are shown in Figure 6. There are 16 local peaks, corresponding to 16 point targets. As shown in Figure 6, the peak positions corresponding to the same target are in the same or adjacent distance units, and they are in different frequency units due to different carrier frequencies. The detection results of the targets are shown in Figure 7. The maximum error of the range is 1.23 m and the average error is 0.52 m. The maximum error of velocity is 0.95 m/s and the average error is 0.36 m/s. Randomly generated 1000 targets for Monte Carlo experiments, the average error of the range is 0.77 m and the average error of the velocity error is 0.04 m/s.

There are eight typical cases of solving Doppler ambiguities for the 16 targets of the above simulation. The Doppler frequencies $f_{D1,amb}$ and $f_{D2,amb}$ (possibly frequency aliasing) are shown in Table 3. The Doppler frequency difference Δf is calculated according to Table 1.

**Figure 8:**
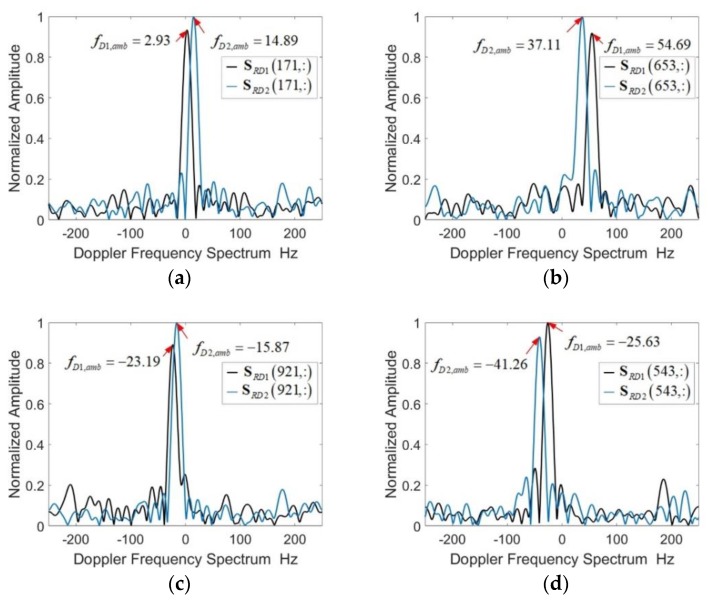

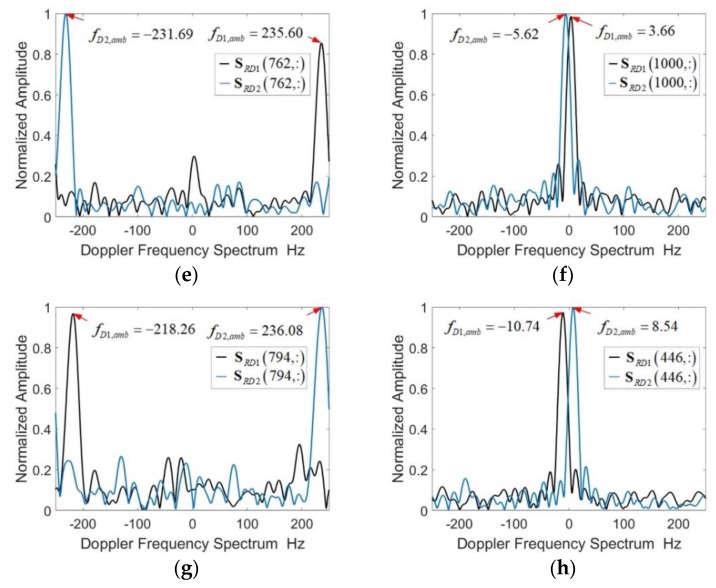
The Doppler spectrum for 8 typical cases. (**a**) $f_{D1,amb}\geq 0$, $f_{D2,amb}\geq 0$, $f_{D1,amb}\leq f_{D2,amb}$ (**b**) $f_{D1,amb}\geq 0$, $f_{D2,amb}\geq 0$, $f_{D1,amb}>f_{D2,amb}$ (**c**) $f_{D1,amb}<0$, $f_{D2,amb}\leq 0$, $f_{D1,amb}<f_{D2,amb}$ (**d**) $f_{D1,amb}<0$, $f_{D2,amb}\leq 0$, $f_{D1,amb}\geq f_{D2,amb}$ (**e**) $f_{D1,amb}\geq 0$, $f_{D2,amb}\leq 0$, $f_{D1,amb}-f_{D2,amb}>f_{D,\max}$ (**f**) $f_{D1,amb}\geq 0$, $f_{D2,amb}\leq 0$, $f_{D1,amb}-f_{D2,amb}<f_{D,\max}$ (**g**) $f_{D1,amb}\leq 0$, $f_{D2,amb}\geq 0$, $f_{D1,amb}-f_{D2,amb}>f_{D,\max}$ (**h**) $f_{D1,amb}\leq 0$, $f_{D2,amb}\geq 0$, $f_{D1,amb}-f_{D2,amb}<f_{D,\max}$.

The eight cases shown in Figure 8 can be roughly divided into two categories according to the velocity direction. The signs of $f_{D1}$ and $f_{D2}$ are the same. When the velocity v<0, then $f_{D2}>f_{D1}>0$. When the velocity v<0, then $f_{D2}<f_{D1}<0$.

Although the signs of $f_{D1,amb}$ and $f_{D2,amb}$ may be different, the numerical relationship of $f_{D1,amb}$ and $f_{D2,amb}$ is consistent with the numerical relationship of $f_{D1}$ and $f_{D2}$. Normally, the number of Doppler frequency aliasing is $q_{2}=q_{1}$.

The velocity of the target (such as vehicles) is less than 50 m/s. According to Equation (19), the Doppler frequency difference Δf is less than 50 Hz. Two special cases shown in Figure 8e,g. When $f_{D1,amb}-f_{D2,amb}>f_{D,\max}$$\left(f_{D1,amb}>0,f_{D2,amb}<0\right)$ or $f_{D2,amb}-f_{D1,amb}>f_{D,\max}$$\left(f_{D2,amb}>0,f_{D1,amb}<0\right)$, it means that the number of Doppler frequency aliasing $q_{2}\neq q_{1}$. When $f_{D1,amb}$ and $f_{D2,amb}$ satisfy the conditions of $f_{D1,amb}-f_{D2,amb}<2f_{D,\max}$ or $f_{D2,amb}-f_{D1,amb}<2f_{D,\max}$, it indicates that $q_{2}=q_{1}+1$.

The velocity direction affects the Doppler frequency aliasing direction. According to the sign and numerical relationship of the Doppler frequency $f_{D1,amb}$ and $f_{D2,amb}$, the velocity direction of the target can be judged, and the aliasing direction can be further judged. Theoretical analysis and simulation results verify the effectiveness of the new radar waveform．

## 5. Conclusions

This paper focuses on the research of continuous wave radar waveform design, analyzes the two-dimensional FFT processing method and the Doppler ambiguity problem for chirp sequence. This paper proposes a new waveform which consists of two chirp sequences, which are intertwined to each other. Each chirp signal has the same frequency modulation, the same bandwidth and the same chirp duration. The carrier frequencies are different and there is a frequency shift which is large enough to ensure that the Doppler frequencies for the same moving target are different. The peak positions in the amplitude spectrums corresponding to the same target are in the same or adjacent distance units, and they are in different frequency units due to different carrier frequencies. According to the sign and numerical relationship of the Doppler frequencies (possibly frequency aliasing), the Doppler frequency ambiguity problem is solved in eight cases.

Theoretical analysis and simulation results verify that the new radar waveform is capable of measuring range and radial velocity simultaneously and unambiguously with high accuracy and resolution even in multi-target situations.

## Figures and Tables

**Table 1:** Discussion on the Doppler frequency difference.

Sign	Numerical Relationship	Doppler Frequency Difference	Velocity	Case
$f_{D1,amb}\geq 0$ $f_{D2,amb}\geq 0$	$f_{D1,amb}\leq f_{D2,amb}$	$q_{2}=q_{1}$,$\Delta f_{D}=f_{D2,amb}-f_{D1,amb}$	v≤0	1
	$f_{D1,amb}>f_{D2,amb}$	$q_{2}=q_{1}$,$\Delta f_{D}=f_{D2,amb}-f_{D1,amb}$	v>0	2
$f_{D1,amb}<0$ $f_{D2,amb}\leq 0$	$f_{D1,amb}<f_{D2,amb}$	$q_{2}=q_{1}$,$\Delta f_{D}=f_{D2,amb}-f_{D1,amb}$	v<0	3
	$f_{D1,amb}\geq f_{D2,amb}$	$q_{2}=q_{1}$,$\Delta f_{D}=f_{D2,amb}-f_{D1,amb}$	v>0	4
$f_{D1,amb}\geq 0$ $f_{D2,amb}\leq 0$	$f_{D1,amb}-f_{D2,amb}>f_{D,\max}$	$q_{2}=q_{1}+1$,$\Delta f_{D}=f_{D2,amb}+2f_{D,\max}-f_{D1,amb}$	v<0	5
	$f_{D1,amb}-f_{D2,amb}<f_{D,\max}$	$q_{2}=q_{1}$,$\Delta f_{D}=f_{D2,amb}-f_{D1,amb}$	v>0	6
$f_{D1,amb}\leq 0$ $f_{D2,amb}\geq 0$	$f_{D2,amb}-f_{D1,amb}>f_{D,\max}$	$q_{2}=q_{1}+1$,$\Delta f_{D}=f_{D2,amb}+\left(-2f_{D,\max}\right)-f_{D1,amb}$	v>0	7
	$f_{D2,amb}-f_{D1,amb}<f_{D,\max}$	$q_{2}=q_{1}$,$\Delta f_{D}=f_{D2,amb}-f_{D1,amb}$	v<0	8

**Table 2:** The basic parameters of the variable carrier frequency chirp sequence.

Parameters	Symbol
The First Carrier Frequency	$f_{01}=24.000\;\mathrm{GHz}$
The Second Carrier Frequency	$f_{02}=24.150\;\mathrm{GHz}$
Sweep Bandwidth	B=100 GHz
The Chirp Duration	$T_{p}=1\;\mathrm{ms}$
Chirp Cycles	L=32
FFT Length for Range Domain	$N_{FFT}=2048$
FFT Length for Doppler Domain	$L_{FFT}=2048$

**Table 3:** Target parameters and measurement results.

Simulation Parameters		Measurement Results					Case
$R_{0}$ m	$v_{0}$ m/s	$f_{D1,amb}\;\mathrm{Hz}$	$f_{D2,amb}\;\mathrm{Hz}$	$\Delta f_{D}\;\mathrm{Hz}$	R m	$v_{r}$ m/s	
7.27	9.37	3.66	−5.62	−9.28	6.94	9.34	6
18.05	−6.12	−23.19	−15.87	7.32	18.06	−6.35	3
31.13	0.00	0.73	0.24	−0.49	31.24	0.00	2
40.65	−32.79	235.60	−231.69	32.71	41.53	−32.70	5
55.15	45.21	−218.26	236.08	−45.66	53.92	45.23	7
67.10	40.00	113.77	71.29	−42.48	66.38	41.08	2
74.75	18.45	54.69	37.11	−17.58	74.32	18.98	2
83.20	−20.00	192.63	213.62	20.99	83.45	−20.54	1
94.86	15.82	−25.63	−41.26	−15.63	94.17	15.37	4
103.44	−18.72	−10.74	8.54	19.28	103.69	−18.82	8
120.23	8.22	187.26	179.20	−8.06	119.88	7.37	2
129.00	22.30	−61.28	−83.74	−22.46	128.22	21.36	4
143.22	14.20	233.15	218.51	−14.64	142.84	13.87	2
156.92	−12.54	2.93	14.89	11.96	157.23	−12.41	1
168.00	17.00	−213.62	−231.69	18.07	167.49	17.30	4
175.00	0.00	0.24	0.24	0.00	174.94	0.00	1
